# Anomaly Detection Method for Industrial Control System Operation Data Based on Time–Frequency Fusion Feature Attention Encoding

**DOI:** 10.3390/s24186131

**Published:** 2024-09-23

**Authors:** Jiayi Liu, Yun Sha, Wenchang Zhang, Yong Yan, Xuejun Liu

**Affiliations:** Information Engineering College, Beijing Institute of Petrochemical Technology, Beijing 102617, China; 2021520129@bipt.edu.cn (J.L.); 2022520251@bipt.edu.cn (W.Z.); yanyong@bipt.edu.cn (Y.Y.); lxj@bipt.edu.cn (X.L.)

**Keywords:** industrial control security, anomaly detection, sensor operation data, feature fusion, attention mechanism

## Abstract

Anomaly detection in industrial control system (ICS) data is one of the key technologies for ensuring the security monitoring of ICSs. ICS data are characterized as complex, multi-dimensional, and long-sequence time-series data that embody ICS business logic. Due to its complex and varying periodic characteristics, as well as the presence of long-distance and misaligned temporal associations among features, current anomaly detection methods in ICS are insufficient for feature extraction. This paper proposes an anomaly detection method named TFANet, based on time–frequency fusion feature attention encoding. Considering that periodic variations are more concentrated in the frequency domain, this method first transforms the time-domain data into the frequency domain, obtaining both amplitude and phase data. Then, these data, together with the original time-series data, are used to extract features from two perspectives: long-term temporal changes and long-distance associations. Finally, the six features learned from both the time and frequency domains are fused, and the feature weights are calculated using an attention mechanism to complete the anomaly classification. In multi-classification tasks on three ICS datasets, the proposed method outperforms three popular time-series models—iTransformer, Crossformer, and TimesNet—across five metrics: accuracy, precision, recall, F1 score, and AUC-ROC, with average improvements of approximately 19%, 37%, 31%, 35%, and 22%, respectively.

## 1. Introduction

As industrial control systems are connected to the Internet, the security of industrial control systems faces severe challenges. Traditional industrial control security research focuses on network protection, which is difficult to deal with once the system is invaded. Regardless of the invasion method, many attacks will cause system failures by tampering with the key data of the industrial control system. Therefore, the anomaly detection of industrial control system operation data is the last line of defense for industrial control security [1]. The anomaly detection of industrial control system operation data refers to judging the security status of the system by calculating whether the operation data of the industrial control system conforms to the normal business logic, so as to promptly alarm dangerous conditions.

ICS data are complex, high-dimensional, long-sequence time-series data that encapsulate the business logic of system operation. ICS data exhibit two distinct characteristics: (1) inconsistent feature periodicity—different features of ICS data are derived from various sensors, each with a unique data variation cycle; and (2) long-distance and misaligned temporal associations—for instance, a change in a flow sensor may lead to a subsequent change in a level sensor. The long-term temporal variation of features is referred to as one perspective, while the associations between features represent another perspective. Due to these characteristics, directly applying popular anomaly detection algorithms in the time-series domain, such as iTransformer, Crossformer, and TimesNet, has not achieved satisfactory results. This is because these methods only extract features based on temporal dependencies among features or use attention mechanisms in isolation, resulting in incomplete feature information extraction from ICS data.

In order to solve the problem of data feature extraction difficulties in industrial control system anomaly detection, this paper proposes a method for industrial control system operation data anomaly detection based on time–frequency fusion feature attention encoding, TFANet. First, the industrial control data are Fourier-transformed to obtain the frequency domain amplitude and phase data of the corresponding data to capture periodic features, thereby improving the problem of feature extraction difficulties caused by period inconsistency; in order to fully learn the long-term changes of features and the long-distance correlation between features, the original time domain data and frequency domain data are extracted separately to obtain six feature maps; in order to mine the deep information of these feature maps, the time domain and frequency domain information are fused, and these features are encoded based on self-attention; finally, a classification module is constructed.

At present, the anomaly detection methods for industrial control system operation data are developed around three types of methods, namely the representation learning of time series data, the feature representation of attention mechanism modeling, and graph neural networks.

Representation learning for time series can be divided into two categories: optimizing the network structure for extracting time series features and optimizing the network loss function. Among the methods for optimizing the feature extraction structure, Sanchez et al. [2] proposed a network structure based on the fusion of variational autoencoders and generative adversarial networks to decouple the temporal relationship of satellite images. The core point is to separate the public information and unique information in the satellite image time series data. Chen et al. [3] proposed a multi-task time series data representation learning method MTRL, which combines a deep wavelet decomposition network and a 1D-CNN residual network to extract multi-scale subsequence features and time domain features, respectively. Franceshi et al. [4] proposed a time series data representation learning framework based on a 1D-CNN encoder network with extended causality to improve the efficiency and scalability of long time series representation learning. Yang and Hong et al. [5] proposed a time series unsupervised representation learning framework BTSF, which is achieved by integrating temporal and spectral information and constructing more reasonable sample pairs. In terms of the integration of temporal and spectral information, an aggregation module is designed to interact with temporal information and spectral information across domains, thereby iteratively extracting features at both temporal and spectral scales. Frankin et al. [6] improved the network based on the TCN architecture [7] and proposed a time embedding module that focuses on capturing time-related features, such as periodicity, trends, and distribution drift. In terms of optimizing network learning methods, Tonekaboni et al. [8] proposed a generative method to decouple global and local feature representations in time series data and use counterfactual regularization to minimize the mutual information between global and local variables. There are two studies that use masked autoencoder architectures, namely TiMAE [9] and SimMTM [10]. Among them, the goal of TiMAE is to solve the problem of distribution drift. Its core is to learn strong representations with less inductive bias. In terms of masking strategy, it creates different views for the encoder at each iteration to make better use of the entire time series input. SimMTM reconstructs the original time series from multiple masked time series through sequence similarity learning and point-level aggregation to implicitly reveal the local structure of the manifold. To this end, it introduces a neighborhood aggregation design for reconstruction, aggregating the point-level representations of the time series by the similarity learned in the series representation space. On top of the reconstruction loss, a constraint loss based on the neighborhood assumption of the time series manifold is proposed to guide sequence representation learning. Among the methods for optimizing network loss functions, contrastive learning has been a relatively popular self-supervised learning method in recent years. The following studies have applied this method to design loss functions: Eldele et al. [11] proposed self-supervised representation learning framework TS-TCC through time series and context contrast. By designing a cross-view prediction task, the enhanced past latent features are used to predict the future of another enhancement at a certain time step, and a new time contrast module is used to learn time dependencies. This module further learns discriminative representations by maximizing the similarity between different contexts of the same sample and minimizing the similarity between different sample contexts. The TNC method [12] applies contrastive learning to focus on complex multivariate non-stationary time series. This method uses debiased contrast loss to ensure that the distribution of signals from the neighborhood and the distribution of non-neighboring signals in the latent space are distinguishable, thereby learning feature representations.

The overall idea of learning methods for time series representation is to model the time-varying features of the original data and to better represent the time-varying features, which effectively improves the representation of feature changes in the depth of time series data feature extraction. However, the value of other features is also ignored. For example, frequency domain data also contain a large number of effective representations of features, and these features can be used to capture the correlation between periodic changes between features.

Related research on feature representation modeling using attention mechanisms includes the iTransformer method proposed by Liu et al. [13], which analyzes the reasons why models based on the Transformer architecture perform poorly on prediction tasks. An important point is that models using this architecture will encode multiple features at the same time as time series tokens at the input stage and then use the attention mechanism to capture the correlation between the moments. However, for all feature points at the same time, the physical meanings they represent are different, and there are large differences in scale. Direct encoding may cause the correlation between features to be eliminated, making it impossible for the model to learn a more efficient representation. To this end, this method encodes a single feature into an independent token without changing the Transformer architecture, so the attention mechanism models the correlation between different features, while the temporal correlation is learned by the feedforward network layer. The TimesNet method proposed by Wu et al. [14] is inspired by the multi-periodicity of time series data. It uses Fourier transform to split the more complex time series feature changes into changes within a single cycle and between multiple cycles. Then, the original one-dimensional time series data are converted into a two-dimensional tensor represented by multiple cycles. Finally, a two-dimensional convolution kernel is used to model and extract the time series changes. The study pointed out that it is complex and tedious to extract periodic changes from multivariate time series data in the time domain alone, but if we focus on the frequency domain, we can use the Fourier transform to obtain periodic frequency changes between different features. Therefore, we first use the Fourier transform to obtain the more obvious periods in the original data and then use these periods to represent the original time domain data, convert the data from one dimension to two-dimensional tensor representation, and finally use two-dimensional convolution to extract the correlation between features. The Crossformer method proposed by Zhang et al. [15] uses the Transformer architecture. Its core point is to model cross-feature dependencies, that is, the correlation between features at different times. The original Transformer’s attention weight distribution focuses on the feature and its adjacent data points and cannot model the attention representation of cross-feature dependencies. On this basis, this method proposes Dimension Segment Wise (DSW) to split the original input into different data segments and then designs a two-stage attention layer to model the attention representation between different data segments. Specifically, the first stage learns the attention representation of different data segments of a single feature to model the dependency relationship across features; the second stage learns the correlation relationship between multiple features. Since the direct application of the attention mechanism will lead to excessive network calculation, this method applies a routing mechanism. First, the input of the first stage is mapped to the routing vector, and then the routing vector distributes the dependency information of the first stage input according to the features so as to construct the correlation relationship across features.

For the method of modeling feature representation using the attention mechanism, industrial control data belong to time series data, which have other characteristics such as high dimensions, long-distance correlation of features, and misaligned time series changes. Although the attention mechanism is good at capturing long-distance correlation, it does not make sufficient use of the temporal position information of the data from the perspective of long time series; from the perspective of long-distance correlation between features, although it can learn the correlation between changes in features, it does not learn enough about the differences in the periodic changes of each feature, resulting in the inability of the anomaly detection model to model a relatively complete feature representation during feature extraction. Therefore, when designing the method, it is necessary to improve the problem of incomplete feature extraction of the anomaly detection model.

The following are some methods for applying graph neural networks: Zhang Zhen et al. [16] proposed the GSC-MAD method, which obtains a stable graph structure under normal conditions and uses the high-dimensional time series embedding representation learned from normal data to achieve the single-step prediction of all variables. Then, the variable behavior deviation reflected by the prediction error is combined with the information propagation deviation between variables reflected by GSC to achieve anomaly detection. Chen et al. [17] proposed the DyGraphAD method. The core idea of this method is to detect outliers based on the relationship between sequences and the deviation of the change of the time pattern within the sequence from the normal state to the abnormal state. At the same time, the evolutionary properties of the graph are used to assist the graph prediction task and the time series prediction task. Zambon et al. [18] proposed a graph data anomaly detection method, GIF. This method considers that some incremental partitions of the data space are easier to isolate anomalies and outliers. Based on the isolation forest method, a new incremental partition of the graph space is introduced, making the isolation forest method applicable to general attribute graphs, that is, graphs where both nodes and edges can be associated with attributes. Zhou LiWen et al. [19] proposed the HAD-MDGAT method, which simultaneously learns the dependencies between sensors in the temporal and spatial dimensions. At the same time, to solve the overfitting problem in the autoencoder-based method, a hybrid GAN and MLP anomaly detection method was proposed. Srinivas et al. [20] proposed the HgAD method, which is used to learn discrete hypergraph structures. In addition, a hierarchical encoder–decoder architecture is used to model the temporal trends and spatial relationships between interdependent sensors to overcome the challenges of high-order dependencies in sensor networks.

The method using graph neural network has shown excellent results in capturing long-distance dependencies between features. By building a graph structure, the model can clearly learn the correlation between features. However, the amount of industrial control time series data is large, and the graph neural network structure is often shallow. Stacking too many layers will cause all vertices to converge. On the other hand, the changes in industrial control time series data are complex, and the graph constructed by the graph neural network cannot change adaptively.

Therefore, considering the advantages and disadvantages of the above three methods, we should start from the characteristics of industrial control data: on the one hand, for the long time series changes and long-distance associations of industrial control data, the time domain features are extracted using the horizontal and vertical dual perspectives; on the other hand, compared with the real domain, signals with similar changes will be expressed with higher concentration in the frequency domain, ignoring the time differences, thereby improving the robustness of the algorithm to the extraction of misaligned time series associations. The above time domain and frequency domain data are brought together to extract data features more completely.

In order to learn the deep information of the time domain and frequency domain data, a time–frequency feature fusion module is proposed. To ensure that the model can fully capture data changes, this module constructs six feature maps from three data samples and the transposition of data samples and uses convolution and other methods to extract features, so as to learn features from two perspectives: the long time series of features and the long-distance association between features. At the same time, to ensure that the model can fully understand the correlation changes between features of different scales, the different scale features output by the six sub-networks are fused into an overall feature map. Finally, a fused feature attention encoding module is proposed. This module uses self-attention encoding to fuse features, calculates attention weights for features of six scales, and uses weights as the basis for anomaly detection, thereby improving the performance of the industrial control data anomaly detection model. The main contributions of this paper are as follows:

1. A method TFANet suitable for the task of anomaly detection of industrial control operation data is proposed. The time–frequency feature fusion module is used to improve the problem of incomplete anomaly detection feature extraction caused by inconsistent industrial control data feature cycles. Compared with the popular time series data anomaly detection method, the performance of the task of anomaly detection of industrial control data is greatly improved.
2. A new perspective for understanding the structure and feature changes of industrial control operation data is proposed, that is, long-distance and dislocated time series association. Through this perspective, the connection between the time series data structure and the business logic of the industrial control system is connected in series, and a new feature extraction method for industrial control time series data is proposed.
3. Experimental validation has shown that when using convolution as the feature extraction method for anomaly detection in ICS data, the start–stop operations of ICSs result in step changes in feature values. Smaller convolutional kernels are more effective in enhancing the overall performance of the model in anomaly detection tasks. The results of relevant ablation experiments further demonstrate that this method can effectively extract features from ICS data.

## 2. Time–Frequency Fusion Feature Attention Encoding

**Figure 1:**
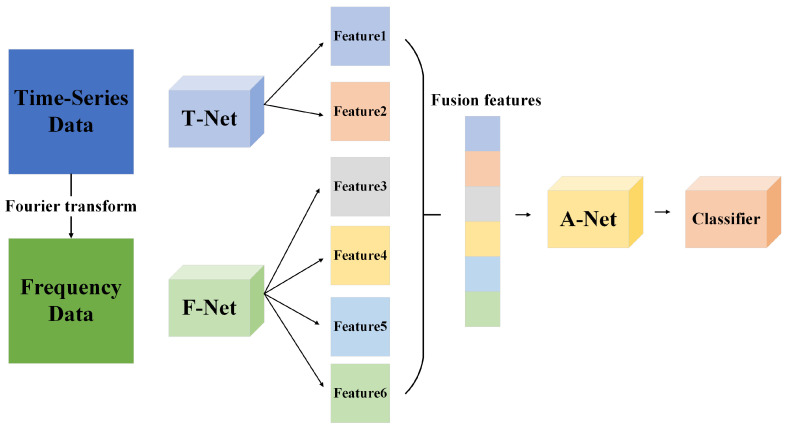
Overall model architecture.

In order to extract feature information from both intra-feature and inter-feature perspectives, a model architecture is designed, as shown in Figure 1. The model as a whole can be divided into three parts, namely T-Net for feature extraction of time domain data, F-Net for the feature extraction of frequency domain data, and A-Net for the attention encoding of time–frequency fusion features.

In the data preprocessing stage, the corresponding frequency domain data can be obtained by Fourier transforming the original industrial control data (time domain data), which contain amplitude and phase.

In the model training phase, the time domain data are input into the T-Net network, and the amplitude data and phase data in the frequency domain are input into the F-Net network. The three types of data are used for feature extraction from two perspectives: long-term time series changes and long-distance associations between features. Finally, six features of different scales are output. The six features are connected and input into the A-Net for attention encoding, that is, the weights of features of different scales are calculated. Finally, the weight calculation results are input into the classifier for anomaly detection tasks.

### 2.1. Time–Frequency Feature Fusion Module

**Figure 2:**
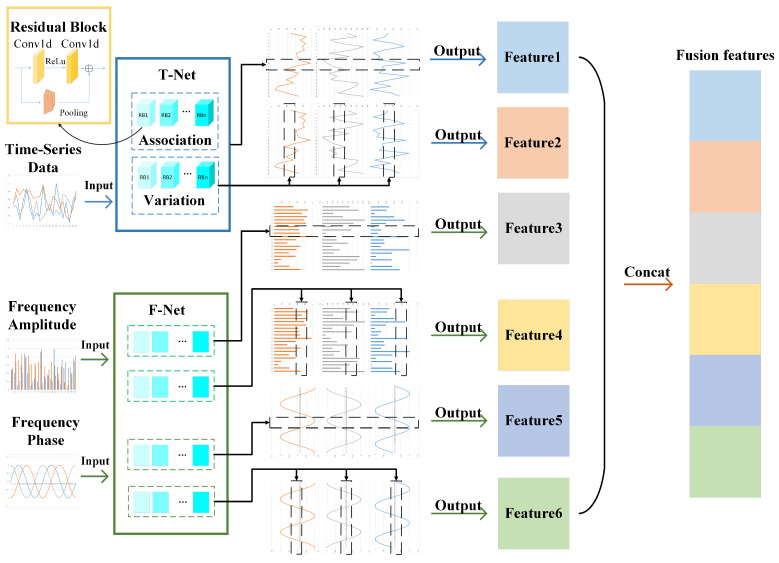
Dual-view time–frequency feature fusion module.

To more closely integrate time-domain and frequency-domain information, a time–frequency feature fusion module was designed based on dual-perspective feature extraction, as shown in Figure 2. The input to this module consists of three different types: time-domain data, frequency-domain amplitude data, and frequency-domain phase data. The two sets of frequency-domain data are obtained by applying a Fourier transform to the time-domain data. This module addresses a series of challenges posed by the characteristics of ICS data by extracting features from both time-domain and frequency-domain data simultaneously. Business logic may be more intuitively represented in the time domain, while certain anomaly patterns might exhibit a more concentrated expression in the frequency domain. By synchronously extracting features from both domains, the model can more accurately capture these potential differences, thereby extracting more comprehensive features from the ICS data.

For time domain data, it is input into T-Net for feature extraction. T-Net consists of two sub-networks, which extract features from two perspectives: long-term temporal changes within features and long-distance correlations between features. Compared with general network structures for time series data such as RNN, LSTM, GRU, etc., which can only extract features from long-term temporal changes of features, this method captures feature changes at another scale, namely, long-distance correlations between features, which enables the model to avoid overfitting caused by a single feature scale.

Some features in industrial control data change relatively slowly, which may cause the gradient vanishing problem in the deep learning model training process. Residual network ResNet is often used to solve the gradient vanishing problem in model training. The method is to pool the original data before and after convolution and add the output after pooling and convolution to retain the gradient. On this basis, the residual block structure shown in the upper left corner of Figure 2 is designed, which consists of two one-dimensional convolutional layers and a pooling layer. The ReLu activation function is added in the middle of the convolutional layer. Multiple residual blocks are superimposed to extract deeper features. If the dual perspective represents feature extraction from breadth, then the stacking of residual blocks is feature extraction from depth. Finally, T-Net outputs the feature extraction results of two different scales. The feature extraction process of T-Net can be expressed as follows. The one-dimensional convolution formula is shown in Equation (1), where $X_{i\times j}$ represents the input data of *i* columns and *j* rows, $W_{c\times i}$ represents the weight matrix, and *c* represents the number of convolution kernels.

(1) $$\mathrm{Conv}\left(X_{i\times j}\right)=\sum_{j=0}^{j}W_{c\times i}\cdot X_{i\times j}$$

Pooling is divided into average pooling and maximum pooling, where *p* represents the padding value on both sides of the input data, *k* represents the number of elements selected for pooling at a time, and *s* represents the step size of pooling. The average pooling calculation formula is shown in Equation (2), and the maximum pooling calculation formula is shown in Equation (3).

(2) $$\mathrm{AvgPool}\left(X_{i\times j}\right)=X_{i\times \lfloor \frac{j+2\times p-k}{s}+1\rfloor }$$

(3) $$\mathrm{MaxPool}\left(X_{i\times j}\right)=X_{i\times \lfloor \frac{j+2\times p-\left(k-1\right)-1}{s}+1\rfloor }$$

The ReLu activation function is shown below Equation (4), where $\max\left(0,j\right)$ represents the larger value between 0 and *j*.

(4) $$\mathrm{ReLu}\left(X_{i\times j}\right)=X_{i\times \max\left(0,j\right)},\;\mathrm{for}\;j=1,2,\ldots ,i$$

Then, the residual block can be expressed as shown in Equation (5).

(5) $$\mathrm{RB}\left(X_{i\times j}\right)=\mathrm{Conv}\left(\mathrm{ReLu}\left(\mathrm{Conv}\left(X_{i\times j}\right)\right)\right)+\mathrm{Pool}\left(X_{i\times j}\right),\mathrm{Pool}\left(X_{i\times j}\right)\in \left\{\mathrm{AvgPool}\left(X_{i\times j}\right),\mathrm{MaxPool}\left(X_{i\times j}\right)\right\}$$

The calculation of the final correlation perspective is shown in Equation (6).

(6) $$\mathrm{PerspH}\left(X_{i\times j}\right)=\sum_{n=1}^{n}{\left(\mathrm{RB}\left(X_{i\times j}\right)\right)}_{n}$$

The calculation formula for the time series perspective is as shown in Equation (7).

(7) $$\mathrm{PerspV}\left(X_{j\times i}\right)=\sum_{n=1}^{n}{\left(\mathrm{RB}\left(X_{j\times i}\right)\right)}_{n}$$

Through Equations (6) and (7), we can obtain the feature matrix output $y_{1},y_{2}$ of T-Net. For frequency domain data, similarly, applying the above sub-network structure to amplitude data and phase data is F-Net. Since two scales of feature extraction are performed for amplitude and phase, respectively, there are four final feature outputs, which respectively represent the amplitude change correlation matrix $y_{3}$ between multiple features, the amplitude change matrix $y_{4}$ of a single feature, the correlation matrix $y_{5}$ of multiple feature periodic changes, and the periodic change matrix $y_{6}$ of a single feature.

After feature extraction by T-Net and F-Net, six features of different scales are obtained. After concatenating the six features, the final fusion feature matrix $y_{fusion}$ is obtained. The process of fusion features can be shown in Equation (8).

(8) $$\mathrm{Concat}\left(y_{1},y_{2},y_{3},y_{4},y_{5},y_{6}\right)=\left[y_{1},y_{2},y_{3},y_{4},y_{5},y_{6}\right]=y_{fusion}$$

### 2.2. Fusion Feature Attention Encoding Module

**Figure 3:**
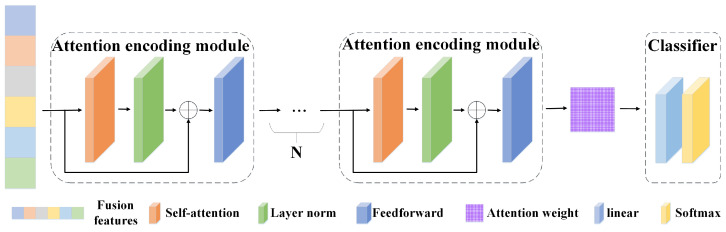
Fusion feature attention encoding module.

In order to learn the extracted features more accurately, a fusion feature attention encoding module is designed. Based on the six concatenated features, this module trains the weights of each feature map according to attention. Figure 3 shows the architecture details of A-Net. A-Net consists of multiple attention encoding modules. This module performs attention calculation on the fusion features output by the time–frequency feature fusion module and outputs the weight values of different features in the fusion features as the basis for downstream anomaly detection tasks.

This module consists of a self-attention layer, layer normalization, a residual structure, and a feedforward layer. The depth can be increased by stacking multiple modules. The calculation of the self-attention layer is mainly performed by Equation (9), where $X_{i}\times j$ represents the input data of *i* columns and *j* rows; $W^{Q},W^{K},W^{V}$ represent the transformation matrices of query, key, and value, respectively; and $d_{K}$ represents the dimension of the input data.

(9) $$\mathrm{SA}\left(X_{i\times j}\right)=\mathrm{softmax}\left(\frac{X_{i\times j}W^{Q}\times X_{i\times j}W^{K}}{\sqrt{d_{K}}}\right)\times X_{i\times j}W^{V}$$

The residual structure first normalizes the output of the self-attention layer and then adds the original self-attention to the normalized result. The calculation is shown in Equation (10). $\mathrm{LN}\left(X_{i\times j}\right)$ represents layer normalization of $X_{i\times j}$.

(10) $$\mathrm{RL}\left(X_{i\times j}\right)=\mathrm{LN}\left(X_{i\times j}\right)+X_{i\times j}$$

The feed-forward layer maps the output of the residual layer to features and then restores the mapping to the original dimension. This design helps the model learn richer feature representations. Its calculation is shown in Equation (11), where $W_{j\times k}$ and $W_{k\times j}$ represent the transformation matrices of *j* columns and *k* rows and *k* columns and *j* rows, respectively.

(11) $$\mathrm{FF}\left(X_{i\times j}\right)=X_{i\times j}\times W_{j\times k}\times W_{k\times j}$$

By integrating Equations (9) to (11), the output of A-Net can be obtained as Equation (12).

(12) $$\mathrm{ANet}\left(X_{i\times j}\right)=\mathrm{FF}(\mathrm{RL}\left(\mathrm{SA}\left(X_{i\times j}\right)\right))$$

During the training phase, the cross entropy is used to calculate the training loss. The training loss L is calculated as shown in Equation (13), where $CLS\left(\cdot \right)$ represents the input classifier.

(13) $$L=\log\left(\frac{\exp\left(\mathrm{CLS}\left(\mathrm{ANet}\left(X_{i\times j}\right)\right)\right)}{\sum_{i=1}^{c}\exp\left(\mathrm{CLS}\left(\mathrm{ANet}\left(X_{i\times j}\right)\right)\right)}\right)$$

The above is the calculation process after the data are input into the fusion feature attention encoding module.

## 3. Experiment and Analysis

### 3.1. Introduction to Dataset

The experiment used two public datasets and one self-built dataset to train and test the model, respectively. The downstream task was set to classify the anomaly and normal data labeled in the dataset.

The SWaT (Secure Water Treatment) dataset [21] is a public industrial control system security research dataset designed to simulate a real water treatment system. The dataset contains data on various types of attacks and normal operations, which are of great significance for evaluating and improving the security of industrial control systems. The dataset contains two categories.

The WADI (Water Distribution Network Dataset) dataset [22] is another public industrial control system security research dataset that simulates a real water supply system. It aims to help researchers and security experts evaluate and improve the security of water supply systems. It contains a large amount of sensor data and network traffic data, which can be used to analyze abnormal behavior and attacks in water supply systems. The dataset contains 14 categories.

**Figure 4:**
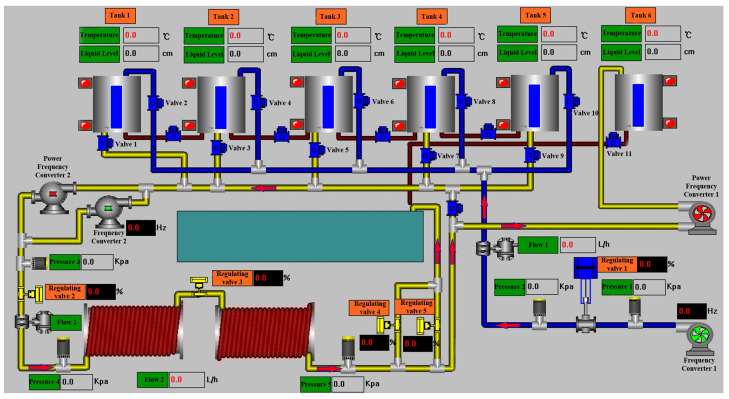
Simulation platform.

The OTS (Oil Transportation Simulation) dataset is a self-built dataset. It was created by simulating a real oil storage business process using a simulation platform equipped with various sensors, in conjunction with a SCADA system model, to construct the business dataset. The simulation platform is shown in Figure 4. The data collection process involved initially operating the simulation system manually under normal conditions and executing business processes to collect normal data. Simultaneously, abnormal data were collected by launching attacks on the system according to its status. Each attack lasted between 1 min and 2 h. During data collection, the following principles were followed: at the start of each collection period, the system was allowed to operate normally for 10 to 15 min. After an attack on the system, the recovery process was delayed by 2 min to 2 h, depending on the impact of the attack. The data collection included attack types that could simulate major accidents in industrial control environments, making such data more prevalent in the dataset. Data from the simulation platform were recorded every 0.5 s. After each round of operation, system data and the timeline were extracted from the system at specified intervals. These principles were implemented to closely replicate the data variation curves of real business scenarios. The final dataset contains 119 features, including sensor values, valve switch states, and certain internal registers of the PLC. A total of 13 types of attacks were simulated, with specific information for each type detailed in Table 1.

When conducting experiments, the data set is divided into different segments and input into the network, and Fourier transform is used to obtain frequency domain data. The following Table 2 shows some data segments, and Table 3 is the result of the Fourier transform of the data in Table 2. For Table 3, the real part of each column of data constitutes the amplitude data, and the imaginary part constitutes the phase data. The final network input is the time domain original data, the amplitude data, and the phase data.

In the data preprocessing stage, the data set is divided into a training set and a test set, and the data in each set are divided into data blocks of fixed length. The data blocks are then Fourier-transformed to obtain amplitude and phase data. In the training stage, the three data blocks are input into TFANet for training. Finally, in the testing stage, the same method is used to perform the anomaly classification task.

### 3.2. Introduction to Evaluation Indicator

Three comparison methods are used, namely iTransformer, TimesNet, and Crossformer. The following five evaluation indicators are used: Accuracy, Precision, Recall, F1-Score, and Area Under the Curve of ROC (ROC-AUC). The values of these five indicators are all between 0 and 1. The higher the score, the better the performance represented by the indicator.

The accuracy rate is used to calculate the ratio of the number of correctly judged samples to the total number of samples. The calculation Equation is shown in the following Equation (14).

(14) $$\mathrm{Accuracy}=\frac{\mathrm{TP}+\mathrm{TN}}{\mathrm{TP}+\mathrm{TN}+\mathrm{FP}+\mathrm{FN}}$$

Among them, the true positive (TP) when the positive sample is predicted as a positive sample, the false negative (FN) when the positive sample is predicted as a negative sample, the false positive class (FP) when the negative sample is predicted as a positive sample, and the true negative class (TN) when the negative sample is predicted as a negative sample.

The precision rate is used to evaluate the proportion of examples that are actually positive samples among those that are classified as positive samples. Its calculation is shown in Equation (15).

(15) $$\mathrm{Precision}=\frac{\mathrm{TP}}{\mathrm{TP}+\mathrm{FP}}$$

The recall rate evaluates the classifier’s ability to identify positive samples, and its calculation is shown in Equation (16).

(16) $$\mathrm{Recall}=\frac{\mathrm{TP}}{\mathrm{TP}+\mathrm{FN}}$$

Generally speaking, the precision and recall cannot be high at the same time. The F1 score is used to comprehensively consider the two. Its calculation is shown in Equation (17).

(17) $$F1=\frac{2\times \mathrm{Precision}\times \mathrm{Recall}}{\mathrm{Precision}+\mathrm{Recall}}$$

The AUC of the multi-classification problem is calculated in the following way: first, for each category, the remaining categories are regarded as negative samples, and the category is regarded as a positive sample. The two indicators of the positive prediction accuracy rate (True Positive Rate, TPR) and the positive prediction error rate (False Positive Rate, FPR) are calculated as follows: Equations (18) and (19).

For each category, the ROC curve of the category is drawn, with the horizontal axis being FPR and the vertical axis being TPR. Then, the area under the ROC curve of each category is calculated, that is, the AUC value of a single category. Finally, the AUC value of each category is averaged as the final AUC score.

(18) $$\mathrm{TPR}=\frac{\mathrm{TP}}{\mathrm{TP}+\mathrm{FN}}$$

(19) $$\mathrm{FPR}=\frac{\mathrm{FP}}{\mathrm{FP}+\mathrm{TN}}$$

The above is the calculation formula for the indicators used in the experiment.

### 3.3. Experiment Result

Relevant experiments were carried out according to the above experimental steps, and the results are shown in Table 4 below. From the data in Table 4, we can see that in terms of accuracy performance, the rankings from high to low are as follows: this method, Crossformer, iTransformer, and TimesNet, which are improved by about 9%, 10%, and 15%, respectively, for the three comparison methods. In terms of precision performance, the rankings from high to low are as follows: this method, iTransformer, TimesNet, and Crossformer, and the maximum improvement of this method in terms of precision improvement is about 41%. In terms of recall performance, the rankings from high to low are as follows: this method, Crossformer, iTransformer, and TimesNet, and the average improvement of this method for the three comparison methods is about 10%. In terms of F1 score performance, the rankings from high to low are as follows: this method, TimesNet, iTransformer, and Crossformer, which are improved by 9%, 11%, and 22%, respectively, for the three methods. In terms of the last item ROC-AUC performance, the rankings from high to low are: this method, iTransformer, Crossformer, and TimesNet, and the average improvement of the three comparison methods is about 10%.

Table 5 below shows the results of experiments on the WADI dataset. From the data in Table 5, we can see that in terms of accuracy performance, the rankings from high to low are as follows: this method, TimesNet, iTransformer, and Crossformer, with the highest improvement reaching 31%. In terms of precision performance, the rankings from high to low are as follows: this method, TimesNet, iTransformer, and Crossformer, and the maximum improvement of this method in terms of precision improvement is about 31%. In terms of recall performance, the rankings from high to low are as follows: this method, TimesNet, iTransformer, and Crossformer, and this method improves by about 14%, 18%, and 31%, respectively, for the three comparison methods, which is similar to the performance in terms of accuracy. On terms of F1 score performance, the rankings from high to low are as follows: this method, TimesNet, iTransformer, and Crossformer, and the maximum improvement of this method in terms of F1 score improvement is about 39%. In terms of ROC-AUC performance, the rankings from high to low are: this method, TimesNet, iTransformer, and Crossformer, and the improvements of the three comparison methods are about 14%, 18%, and 31%, respectively.

Table 6 below shows the results of the experiment on the OTS dataset. From the data in Table 6, we can see that in terms of accuracy, the ranking from high to low is as follows this method, Crossformer, TimesNet, and iTransformer, and the average improvement for the three methods is about 26%. In terms of accuracy, recall, and F1 score, this method has a greater improvement than the three methods. In terms of ROC-AUC performance, the ranking from high to low is: this method, TimesNet, Crossformer, and iTransformer, and the average improvement for the three methods is about 23%. According to the above experimental results, TFANet has achieved different degrees of improvement in indicators compared with the other three methods.

The experimental results of the three comparison methods are analyzed below.

(1) For the iTransformer method, compared with the original Transformer model, this method uses the feature column as the query object and compares the correlation between the feature columns. The advantage of this method is that the optimized model can more fully learn the correlation representation of different variables of the input data for the attention layer of the original Transformer model, that is, learn the long-distance correlation between features. However, each column feature of industrial control data has its own unique change process, reflecting the characteristics of different sensor data. The use of a single embedding layer may not be able to encode each change process, and the above-mentioned misaligned time series phenomenon is also reflected in the change process of the feature, resulting in information loss in the time domain, affecting the classification results.

(2) For the Crossformer method, this method proposes the concept of cross-dimensional dependency based on the Transformer model to better capture the correlation between different dimensions in multivariate time series data. Specifically, unlike iTransformer, which embeds each feature column, Crossformer splits the time domain data of each feature column into different data segments for embedding and then calculates attention for different data segments of different feature columns. This attention is named cross attention. This method solves the impact of misaligned time series to a certain extent and highlights the importance of local features. However, for industrial control operation data anomaly detection, the global features of a single variable also contain certain business logic. Splitting data segments on different data sets may destroy the overall business logic and reduce the accuracy.

(3) For the TimesNet method, the core of this method is to capture the correlation changes between different periods of time series data and use this as the basis for downstream tasks. In terms of specific implementation, TimesNet obtains period information through Fourier transform, extracts the correlation of time domain data represented by different periods through convolution, and then fuses it with the original data for downstream tasks. For the anomaly detection of industrial control operation data, this method can capture the cross-period correlation of sensor data in different periods and has better downstream task generalization than iTransformer and Crossformer. However, the selection of the maximum period in this method will be affected by different data sets. For those with more obvious period changes, relatively good results can be achieved.

The two modules of this method are designed according to the characteristics of industrial control data. In the feature fusion module, features are extracted from the perspectives of time series and association, as well as the time domain and frequency domain, to obtain the correlation between variables and time series changes. At the same time, in the frequency domain, the period of each feature column is characterized by the data changes of the two attributes of amplitude and frequency, which improves the problem of period differences between features and has better robustness to misaligned time series. The above experimental results also illustrate the effectiveness of this method to a certain extent.

## 4. Ablation Study

To further explore the impact of the size of the convolution kernel of the one-dimensional convolution layer in T-Net and F-Net, the number of attention layers and the dimension of the feedforward layer in A-Net on the model detection results, the following ablation experiments were conducted using the OTS dataset: the convolution kernel size was set to 1 × 3 and 1 × 11; the number of attention layers in A-Net was set from no attention layer (zero layers) to four attention layers, and two setting strategies were adopted. One was the strategy of increasing the dimension of the feedforward layer: when the number of attention layers was set to 0, 1, 2, 3, and 4, the dimensions of the feedforward layer were 32, 64, 128, and 256, respectively; the other was the strategy of reducing the dimension of the feedforward layer: when the number of attention layers was set from 0 to 4, the dimensions of the feedforward layer were 1024, 512, 256, and 128, respectively. The experimental results of the attention layer increase and dimension increase strategies are shown in Table 7 below.

**Figure 5:**
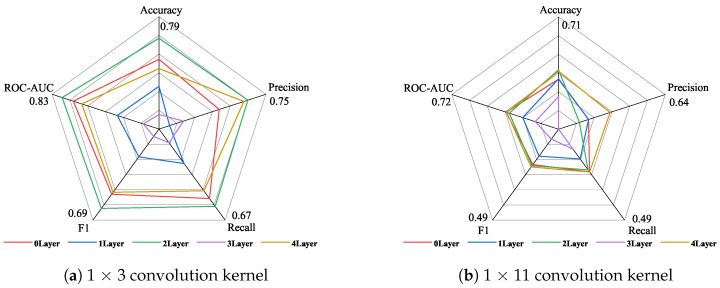
The performance of different convolution kernels under the strategy of increasing the attention layer and increasing the dimensions.

The data in Table 7 are plotted as a radar chart in Figure 5 below to show the different performances of the 1 × 3 small convolution kernel and the 1 × 11 large convolution kernel. Figure 5a shows the specific indicators of using the 1 × 3 convolution kernel, and Figure 5b shows the indicators of using the 1 × 11 large convolution kernel. As can be seen from Figure 5a, when using the 1 × 3 convolution kernel and the dimension increase strategy, setting two attention layers achieves the best results, and the highest values are achieved in all five indicators; setting three attention layers achieves the worst results, and the lowest values are achieved in four indicators. As can be seen from Figure 5b: when using the 1 × 11 convolution kernel, setting four attention layers achieves the best results, and setting three attention layers achieves the worst results.

Comparing the experimental results shown in Figure 5a,b, it is found that setting the one-dimensional convolution layer as a 1 × 3 convolution kernel can achieve better results than the 1 × 11 convolution kernel under the dimension increase strategy. Figure 5 is drawn according to the data in Table 7 to observe the improvement of the five indicators using the 1 × 3 convolution kernel compared with the 1 × 11 convolution kernel under the dimension increase strategy.

**Figure 6:**
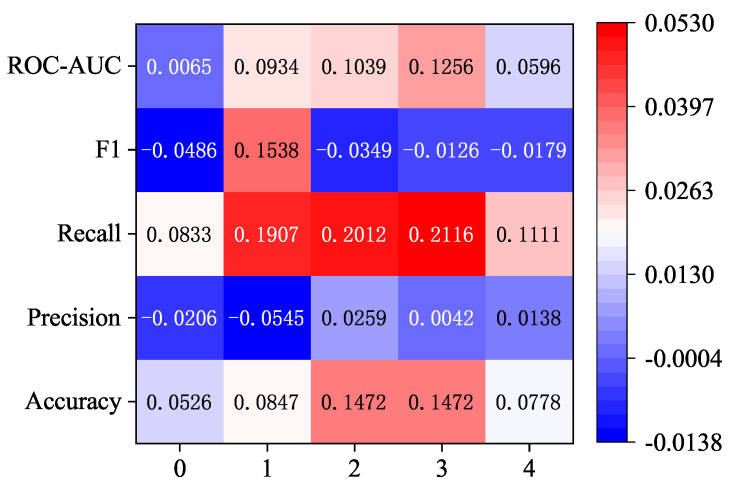
Improvement results of 1 × 3 convolution kernel compared with 1 × 11 convolution kernel under the strategy of increasing the attention layer and increasing the dimension.

As can be seen from Figure 6, under the same attention layer, the use of the 1 × 3 convolution kernel has different degrees of improvement on the five indicators, among which the recall rate is more obviously improved, and it has a certain degree of improvement compared with other indicators when one, two, and three layers of attention are compared. At the same time, there are also some indicators that are reduced, such as F1 scores at 0, 2, 3, and 4 layers of attention and precision at 0 and 1 layers of attention.

In summary, overall, using the 1 × 3 convolution kernel can achieve better results than the 1 × 11 convolution kernel under the dimension increase strategy.

**Figure 7:**
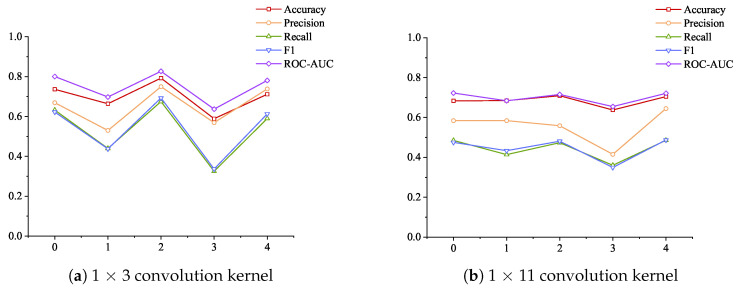
The indicators of different convolution kernels change with the number of layers and dimensions under the strategy of increasing the number of attention layers and increasing the dimensions.

Considering the impact of increasing the number of attention layers on the model detection results, the data in Table 7 plotted the changes in the performance of different convolution kernels under the strategy of increasing the number of attention layers and increasing the dimension, as shown in Figure 7. As the number of attention layers increases and the dimension continues to increase, the use of 1 × 3 convolution kernels shows tortuous changes in the five indicators, and the use of 1 × 11 convolution kernels also shows tortuous changes in the five indicators. From the overall trend, the changes in the three indicators of recall rate, F1 score, and ROC-AOC are similar to those of 1 × 3 convolution kernels but more gentle. The change in precision rate shows a decrease first and then an increase, and the maximum value is achieved at four attention layers. The change in accuracy rate gradually increases from no attention layer to 2 attention layers to reach the maximum value and then begins to decrease and then increase.

There are many start–stop operations in industrial control systems, which reflects the “instantaneous jump” in data changes. The results shown in Figure 7 show that the use of a 1 × 3 small convolution kernel is better at extracting jump features, so the overall accuracy is better. The increase in the number of attention layers has little effect on the accuracy of large convolution kernels, indicating that large convolution kernels are not very sensitive to features. Although the change in the number of attention layers has a large impact on the final index when a small convolution kernel is selected, the average value of all evaluation results exceeds the above three comparison algorithms, which also shows the stability of the method proposed in the paper.

The experimental results under the strategy of increasing the attention layer and reducing the dimension are shown in Table 8.

**Figure 8:**
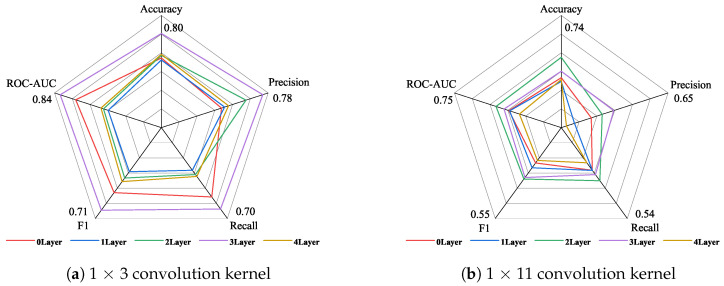
The performance of different convolution kernels under the strategy of increasing the attention layer and decreasing the dimensions.

Similarly, the data in Table 8 are plotted as a radar chart, as shown in Figure 8, to observe the performance of different convolution kernels under the dimensionality-reduction strategy. As shown in Figure 8a, the best performance is achieved when three attention layers are set when using the 1 × 3 convolution kernel, and the worst performance is achieved when one attention layer is set. As shown in Figure 8b, when using the 1 × 11 convolution kernel, the best comprehensive effect is achieved when two attention layers are set, and the lowest indicator evaluation is achieved when four attention layers are set. Comparing the performance of the five indicators of the 1 × 3 convolution kernel and the 1 × 11 convolution kernel, the highest indicator result of the 1 × 3 convolution kernel is higher than the highest indicator result of the 1 × 11 convolution kernel.

**Figure 9:**
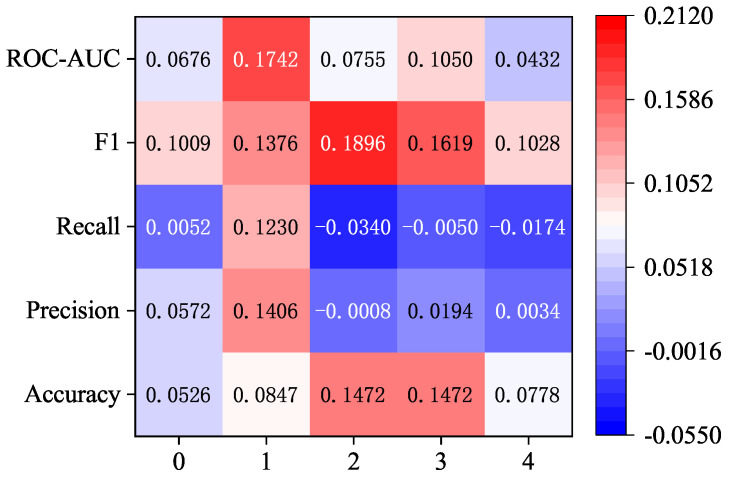
Improvement results of 1 × 3 convolution kernel compared with 1 × 11 convolution kernel under the strategy of increasing the attention layer and decreasing the dimensionality.

Figure 9 shows the improvement in the indicators from using 1 × 3 convolution kernels over 1 × 11 convolution kernels under the dimension reduction strategy. As shown in Figure 8, the recall rate indicator of using 1 × 3 convolution kernels is reduced compared with that of using 1 × 11 convolution kernels, which is mainly reflected in the setting of two, three, and four attention layers. The precision indicator of setting two attention layers also shows a decline. In addition to the above two indicators, the other three indicators have increased to varying degrees. Among them, the most obvious improvement is the ROC-AUC score under setting one attention layer and the F1 score under setting two attention layers, which are increased by about 0.1742 and 0.1896, respectively.

**Figure 10:**
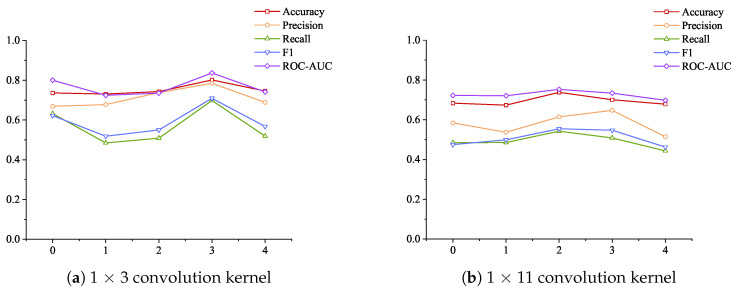
The indicators of different convolution kernels change with the number of layers and dimensions under the strategy of increasing the number of attention layers and decreasing the dimension.

Figure 10 is drawn from the data in Table 8 to more intuitively observe the changes in indicators of different convolution kernels under the strategy of increasing the attention layers and reducing the dimensions. By comparing Figure 10a,b, it can be seen that the increase in the number of layers has a greater impact on the indicator results under the 1 × 3 small convolution kernel.

The above ablation experiments show the influence of three parameters, namely the size of the convolution kernel, the number of attention layers, and the dimension of the feedforward layer, on the model detection results. Combined with the results of the above ablation experiments, the following conclusions can be drawn for the method proposed in this paper: (1) Using a 1 × 3 convolution kernel in the industrial control data anomaly detection task can improve the detection effect of the model to a certain extent compared with using a 1 × 11 convolution kernel. (2) Changes in the number of attention layers and the dimension of the feedforward layer have little effect on the detection results of the model using a large 1 × 11 convolution kernel.

As for conclusion (1), it can be seen from the results of Figure 4 and Figure 7 that, regardless of whether the dimension is increased or decreased, the model using the 1 × 3 convolution kernel achieves the highest values of the five indicators compared with the 1 × 11 convolution kernel. First, from the perspective of the size of the convolution kernel, the 1 × 3 convolution kernel may be more sensitive to the local feature changes in the input data. The misaligned timing phenomenon in the operation of the industrial control system is reflected in the industrial control data as the jump of the data at the next moment. Therefore, using a small convolution kernel can better capture the sharp changes in different feature columns of the two samples. Secondly, from the perspective of the function of the convolution kernel, using a smaller convolution kernel can provide more complex nonlinear transformations, which is conducive to building a deeper network and enhancing the network’s ability to express features. Finally, from the perspective of the network structure, according to the method in this paper, the features extracted by the small convolution kernel will be fused and input into A-Net for attention encoding. The use of a small convolution kernel can more effectively retain the local feature changes in the time domain and frequency domain. In the calculation of attention weights, the differences in feature changes in different domains can be highlighted, and the calculation accuracy of weights can be improved.

As for conclusion (2), also from the perspective of convolution kernel size, as the convolution kernel increases, the receptive field also increases. Therefore, a large convolution kernel is better at extracting global features. When processing data jumps are caused by system start–stop operations, an overly large receptive field may focus on capturing the overall feature changes in the data and cause information loss of feature changes between samples. The main task of the T-Net and F-Net sub-networks of the model is to capture the jumps in feature values. If the lost information contains this important feature, then in the subsequent attention weight calculation, the A-Net sub-network’s weight calculations for the time domain and frequency domain may tend to be averaged. At this time, even changing the number of attention layers cannot make up for the impact of information loss.

In summary, for the method proposed in this article, the use of a small 1 × 3 convolution kernel is more sensitive to the feature changes caused by the misaligned timing phenomenon and can improve the model detection accuracy compared to a large 1 × 11 convolution kernel. Therefore, when constructing the model proposed by the method in this article, it is better to consider using a small convolution kernel similar to 1 × 3.

Table 9 shows the results of the ablation experiment using a 1 × 3 convolution kernel to fix the dimension size of the feedforward layer. The purpose of this experiment is to explore the impact of the dimension increase or dimension reduction strategy. The experimental settings are as follows: T-Net and F-Net use 1 × 3 convolution kernels. When adding attention layers to A-Net, the dimension of the feedforward layer is fixed in size and no longer increases or decreases with the number of layers. The values are set to 32, 64, 128, 256, 512, and 1024. The number of attention layers is set to 0, 1, 2, 3, and 4.

**Figure 11:**
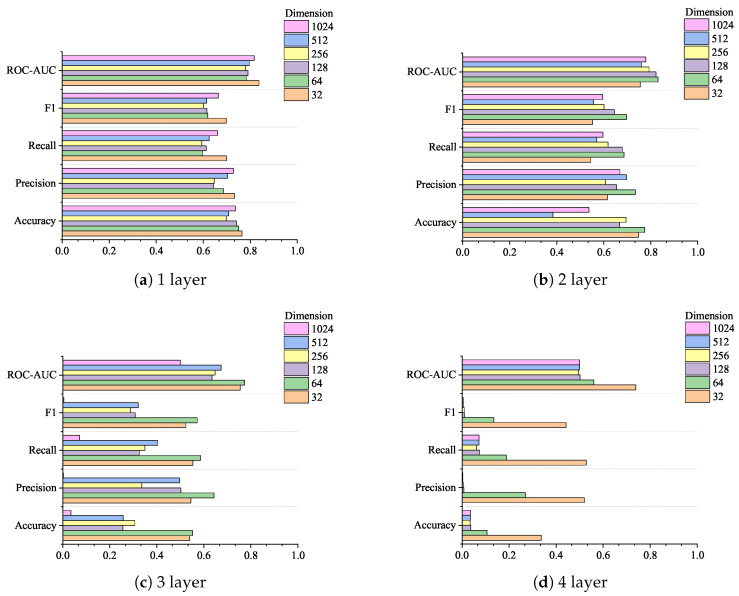
Comparison of model detection index results under different attention layers.

Figure 11 shows a comparison of model detection indicators under different numbers of attention layers. (a), (b), (c), and (d) represent the performance of the five indicators at different feedforward layer dimensions when the attention layers are 1, 2, 3, and 4, respectively. From the information in the figure, we can see that the indicator results are the worst when the attention layers are 4, and the indicator results are higher than 4 when the attention layers are 3. At the same time, by comparing (c) and (d), we can see that the remaining dimensions of the feedforward layer with dimensions higher than 32 have all experienced a significant decline in indicator performance. This may be due to information dilution. By adding attention layers, the model’s ability to perform complex features can be improved, but a network that is too deep may dilute the feature information, causing the model to experience gradient disappearance during training.

**Figure 12:**
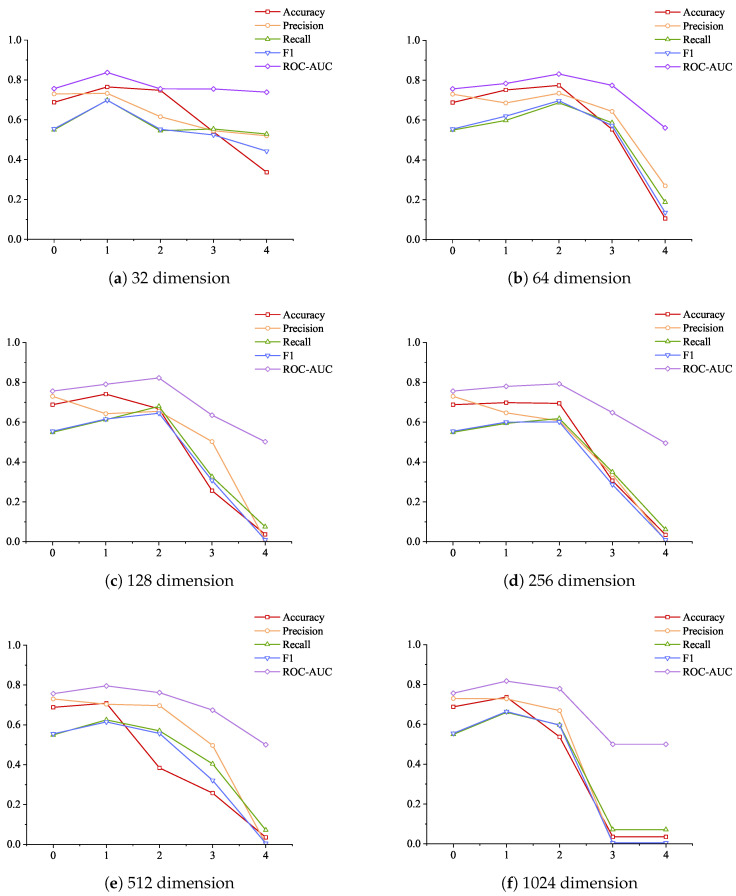
Changes in model detection indicators under different feedforward layer dimensions as the number of layers increases.

In order to more intuitively reflect the changes in the indicators of the model with the increase in the number of layers under different feedforward layer dimension parameters, Figure 12 is drawn, where (a), (b), (c), (d), (e), and (f) represent the indicator changes under 32, 64, 128, 256, 512, and 1024 dimensions, respectively. As can be seen from the figure, most of the indicator change trends of (b), (c), (d), and (f) are consistent with the trend of decreasing from the non-attention layer to the two-layer attention layer, among which the decreasing trend of (f) is the most dramatic. The feedforward layer is usually used to map features to higher dimensions for learning more complex feature relationships. However, this is also accompanied by a series of problems caused by the increase in dimensions. The process of mapping the original features to high-dimensional space and then restoring them to the original dimensions may discard part of the original features. The industrial control data reflect the changes in different sensors of the industrial control system over time, and there is a correlation between each sensor. If this part of the feature changes is discarded during the mapping process, the model will not be able to effectively identify the anomaly, resulting in a decrease in the indicator results.

From the above experiments, we can see that when determining the number of attention layers and the dimension of the feedforward layer in the A-Net sub-network of this method, it is necessary to consider the negative impact of the increase in the number of layers and the increase in dimension. As shown by the experimental results, choosing three attention layers and a dimensionality reduction strategy can achieve the best results.

## 5. Discussion and Conclusions

This paper proposes a method for detecting anomalies in industrial control operation data based on time–frequency fusion feature attention coding, TFANet. This method integrates original time domain data, frequency domain amplitude, and phase data and extracts features from two perspectives: long-term temporal changes within the sample features of industrial control data and long-distance correlations between features. The weights of different output features are calculated through attention coding, which is used as the basis for the task of detecting anomalies in industrial control operation data. After being verified on two public datasets and one self-built dataset, this method outperforms three other popular time series data anomaly detection methods, iTransformer, TimesNet, and Crossformer, in three datasets, proving the effectiveness of this method.

Additionally, ablation experiments on the convolution kernel size in the T-Net and F-Net sub-networks, as well as the number of attention layers and the feedforward layer dimensions in the A-Net sub-network, demonstrated that using smaller convolution kernels can better capture feature variations in ICS anomaly detection tasks, further validating the effectiveness of the proposed method. Specifically, the dataset used in this study consists of ICS sensor operation data, which encapsulates the business logic of ICS operation. For anomaly detection tasks, capturing these patterns is beneficial for extracting more in-depth feature information. Based on all ablation experiment results, an important conclusion is that both local and global feature information play a simultaneous role in anomaly detection. In the OTS dataset constructed in this paper, certain start–stop operations are part of the business logic in ICS data. This “pattern” manifests as instantaneous jumps in the data. To capture such abrupt changes, increasing the weight of local information, i.e., using a smaller convolution kernel (a 1 × 3 kernel was used in this study), may be more advantageous. On the other hand, global information might be more effective in capturing other “patterns” that represent the remaining business logic. Therefore, when applying this method to other datasets, factors such as anomaly types, system business logic, and data dimensions need to be considered to define the specific size of the convolution kernel.

## Figures and Tables

**Table 1:** Attack type details.

Attack Type	Represents Abnormal Events
1	Abnormal decrease in real-time data from the pump pressure sensor may cause the oil transfer process to stop.
2	Abnormal increase in real-time data from the pump pressure sensor may cause the oil transfer process to stop.
3	The valve is abnormally closed during oil transportation.
4	The tank level has been tampered with and exceeds the set maximum value.
5	The tank level has been tampered with and is below the set minimum value.
6	The oil tank collection process is interrupted by the increase in the range and directly enters the sedimentation process.
7	The oil tank collection process was interrupted by the reduction of the range, and the process flow was changed to collect oil in preparation.
8	The set value increases, the oil tank sedimentation process is interrupted, affecting the oil quality.
9	The set value is reduced, the oil tank sedimentation process is prolonged, and the oil tank cannot deliver oil in time.
10	The pipeline pressure alarm value under normal process flow is tampered with, and an abnormal alarm occurs.
11	Under normal process flow, the oil tank temperature alarm value is tampered with, and an abnormal alarm occurs.
12	Hijacking data packets and filling PLC with “0” data, the system stops working.
13	An error instruction is sent to the PLC start/stop core, causing the system to shut down abnormally.

**Table 2:** Time domain data fragment.

Sensor 1	Sensor 2	Sensor 3	Sensor 4
24.64	24.54	20.8912	24.0741
24.64	24.54	20.8333	24.0162
24.64	24.56	20.8333	24.0162
24.64	24.52	20.8333	24.0162
24.64	24.46	20.8333	24.0162
24.64	24.54	20.8333	24.0162

**Table 3:** Frequency domain data fragment.

Sensor 1	Sensor 2	Sensor 3	Sensor 4
147.840 + 0.000j	147.160 + 0.000j	0.013 + 0.000j	0.014 + 0.000j
0.000 − 0.000j	0.050 − 0.087j	0.058 − 0.000j	0.058 − 0.000j
0.000 + 0.000j	0.010 + 0.087j	0.058 + 0.000j	0.058 + 0.000j
0.000 + 0.000j	0.040 + 0.000j	0.058 + 0.000j	0.058 + 0.000j
0.000 − 0.000j	0.010 − 0.087j	0.058 − 0.000j	0.058 − 0.000j
0.000 + 0.000j	0.050 + 0.087j	0.058 + 0.000j	0.058 + 0.000j

**Table 4:** SWaT dataset experimental results.

Method	Accuracy	Precision	Recall	F1-Score	ROC-AUC
iTransformer	0.52023	0.49901	0.49939	0.45572	0.49939
TimesNet	0.47252	0.47257	0.47244	0.47196	0.47244
Crossformer	0.53332	0.26666	0.50000	0.34782	0.50000
TFANet	0.62048	0.67495	0.59892	0.56257	0.59892

**Table 5:** WADI dataset experimental results.

Method	Accuracy	Precision	Recall	F1-Score	ROC-AUC
iTransformer	0.62757	0.78787	0.62355	0.56346	0.62351
TimesNet	0.67224	0.80329	0.66862	0.62975	0.66862
Crossformer	0.49576	0.49981	0.49992	0.41065	0.49992
TFANet	0.80152	0.80743	0.80256	0.80089	0.80254

**Table 6:** OTS dataset experimental results.

Method	Accuracy	Precision	Recall	F1-Score	ROC-AUC
iTransformer	0.51301	0.05357	0.06615	0.05609	0.49730
TimesNet	0.52446	0.04547	0.07677	0.05486	0.50321
Crossformer	0.59851	0.04276	0.07143	0.05349	0.50001
TFANet	0.80160	0.78486	0.69823	0.70926	0.83673

**Table 7:** Experimental results under the strategy of increasing the attention layer and increasing the dimensions.

Layer	Kernel-Size	Accuracy	Precision	Recall	F1-Score	ROC-AUC
	1 × 3	0.73613	0.66899	0.63189	0.62233	0.80032
0	1 × 11	0.68355	0.58424	0.48473	0.47514	0.72257
	AVG	0.70984	0.62662	0.55831	0.54874	0.76145
	1 × 3	0.66394	0.52971	0.43956	0.43757	0.69767
1	1 × 11	0.68457	0.58425	0.41368	0.43339	0.68390
	AVG	0.67426	0.55698	0.42662	0.43548	0.69079
	1 × 3	0.79235	0.74934	0.67479	0.69260	0.82648
2	1 × 11	0.70908	0.55866	0.47359	0.48100	0.71543
	AVG	0.75072	0.65400	0.57419	0.58680	0.77096
	1 × 3	0.58883	0.56896	0.32521	0.33644	0.63672
3	1 × 11	0.63747	0.41512	0.36016	0.34905	0.65462
	AVG	0.61315	0.49204	0.34269	0.34275	0.64567
	1 × 3	0.71138	0.73815	0.58949	0.61273	0.78079
4	1 × 11	0.70485	0.64474	0.48554	0.48708	0.72120
	AVG	0.70812	0.69145	0.53752	0.54991	0.75100

**Table 8:** Experimental results under the strategy of increasing the attention layer and decreasing the dimensions.

Layer	Kernel-Size	Accuracy	Precision	Recall	F1-Score	ROC-AUC
	1 × 3	0.73613	0.66899	0.63189	0.62233	0.80032
0	1 × 11	0.68355	0.58424	0.48473	0.47514	0.72257
	AVG	0.70984	0.62662	0.55831	0.54874	0.76145
	1 × 3	0.73080	0.67771	0.48478	0.51853	0.72435
1	1 × 11	0.67359	0.53712	0.48561	0.49908	0.72092
	AVG	0.70220	0.60742	0.48520	0.50881	0.72264
	1 × 3	0.74310	0.73779	0.50847	0.54982	0.73540
2	1 × 11	0.73791	0.61481	0.54245	0.55485	0.75275
	AVG	0.74051	0.67630	0.52546	0.55234	0.74408
	1 × 3	0.80160	0.78485	0.69823	0.70926	0.83673
3	1 × 11	0.70073	0.64725	0.50866	0.54732	0.7339
	AVG	0.75117	0.71605	0.60345	0.62829	0.78532
	1 × 3	0.74597	0.68874	0.51894	0.56755	0.74121
4	1 × 11	0.67841	0.51449	0.44347	0.46259	0.69800
	AVG	0.71219	0.60162	0.48121	0.51507	0.71961

**Table 9:** 1 × 3 convolution kernel fixed dimension layer experimental result data.

Layer	Dimension	Accuracy	Precision	Recall	F1-Score	ROC-AUC
0	/	0.68779	0.72985	0.54948	0.55453	0.75626
	32	0.76505	0.73269	0.69866	0.69772	0.83729
	64	0.75107	0.68552	0.59814	0.61939	0.78337
	128	0.74098	0.64270	0.61241	0.61560	0.79023
1	256	0.69808	0.64693	0.59406	0.60032	0.77951
	512	0.70852	0.70355	0.62467	0.61456	0.79567
	1024	0.73676	0.72804	0.66015	0.66403	0.81678
	32	0.74780	0.61544	0.54508	0.55211	0.75527
	64	0.77397	0.73425	0.68655	0.69675	0.83116
	128	0.66713	0.65430	0.67918	0.6449	0.82224
2	256	0.69460	0.60695	0.61814	0.60129	0.79265
	512	0.38370	0.69636	0.56986	0.55656	0.76145
	1024	0.53686	0.66885	0.59669	0.59529	0.77883
	32	0.53956	0.54506	0.55379	0.52386	0.75484
	64	0.55311	0.64338	0.5861	0.57212	0.77421
	128	0.25637	0.50277	0.32647	0.3079	0.63528
3	256	0.30652	0.33648	0.34987	0.28695	0.64794
	512	0.25718	0.49665	0.40394	0.32127	0.67393
	1024	0.03507	0.00250	0.07143	0.00484	0.5000
	32	0.33663	0.51943	0.52851	0.44238	0.73881
	64	0.10586	0.26915	0.18766	0.13538	0.56070
	128	0.03677	0.00784	0.07514	0.00931	0.50192
4	256	0.03424	0.00487	0.06134	0.00893	0.49467
	512	0.03507	0.00250	0.07143	0.00484	0.50000
	1024	0.03507	0.00250	0.07143	0.00484	0.50000

## Data Availability

The WADI dataset analyzed during the current study is available at https://itrust.sutd.edu.sg/, accessed on 1 November 2023. The SWaT dataset analyzed during the current study is available at https://itrust.sutd.edu.sg/itrust-labs_datasets/dataset_info/, accessed on 1 November 2023. Other datasets are available from the corresponding author, Yun Sha, upon reasonable request.
